# Clinical impact of diabetes mellitus on 2-year clinical outcomes following PCI with second-generation drug-eluting stents; Landmark analysis findings from patient registry: Pooled analysis of the Korean multicenter drug-eluting stent registry

**DOI:** 10.1371/journal.pone.0234362

**Published:** 2020-06-10

**Authors:** Cheol Hyun Lee, Sang-Woong Choi, Seung-Woon Jun, Jongmin Hwang, In-Cheol Kim, Yun-Kyeong Cho, Hyoung-Seob Park, Hyuck-Jun Yoon, Hyungseop Kim, Chang-Wook Nam, Seongwook Han, Kwon-Bae Kim, Seung-Ho Hur

**Affiliations:** Division of Cardiology, Department of Internal Medicine, Keimyung University Dongsan Hospital, Daegu, South Korea; University of Bologna, ITALY

## Abstract

**Background:**

Patients with diabetes mellitus are at an increased risk for adverse clinical events following percutaneous coronary interventions (PCI). However, the clinical impact of diabetes mellitus (DM) on second-generation drug-eluting stent (DES) implantation is not well-known. The aim of the current analysis was to examine the clinical impact of DM on clinical outcomes and the time sequence of associated risks in patients treated with second-generation DES.

**Methods:**

Using patient-level data from two stent-specific, all-comer, prospective DES registries, we evaluated 1,913 patients who underwent PCI with second-generation DES between Feb 2009 and Dec 2013. The primary outcomes assessed were two-year major cardiac adverse events (MACE), composite endpoints of death from any cause, myocardial infarction (MI), and any repeat revascularization. We classified 0–1 year as the early period and 1–2 years as the late period. Landmark analyses were performed according to diabetes mellitus status.

**Results:**

There were 1,913 patients with 2,614 lesions included in the pooled dataset. The median duration of clinical follow-up in the overall population was 2.0 years (interquartile range 1.9–2.1). Patients with DM had more cardiovascular risk factors than patients without DM. In multivariate analyses, the presence of DM and renal failure were strong predictors of MACE and target-vessel revascularization (TVR). After inverse probability of treatment weighting (IPTW) analyses, patients with DM had significantly increased rates of 2-year MACE (HR 2.07, 95% CI; 1.50–2.86; P <0.001). In landmark analyses, patients with DM had significantly higher rates of MACE in the early period (0–1 year) (HR 3.04, 95% CI; 1.97–4.68; P < 0.001) after IPTW adjustment, but these findings or trends were not observed in the late period (1–2 year) (HR 1.24, 95% CI; 0.74–2.07; P = 0.41).

**Conclusions:**

In the second-generation DES era, the clinical impact of DM significantly increased the 2-year event rate of MACE, mainly caused by clinical events in the early period (0–1 year). Careful observation of patients with DM is advised in the early period following PCI with second-generation DES.

## Introduction

Previous studies have shown that percutaneous coronary interventions (PCI) with drug-eluting stent (DES) has a better outcome than bare-metal stents in patients with diabetes mellitus (DM) [1–4]. Two large randomized trials showed that second-generation DES outperformed first-generation DES by reducing target lesion revascularization (TLR), target vessel revascularization (TVR), and stent thrombosis (ST). But, these improvement of device-oriented clinical outcomes between first- and second-generation DES were seen only in patients without DM and not in patients with DM [5, 6]. DM still remains associated with an increased risk of in-stent restenosis, TLR, or TVR in patients undergoing PCI [7]. However, the overall clinical outcomes, early (0–1 year) and late period (>1 year) efficacies and safety of second-generation DES in DM patients remain controversial. Therefore, to compare the overall clinical outcomes and time sequence of efficacy and safety of two second-generation DES (everolimus-eluting stent (EES) and zotalolimus-eluting stent (ZES)) in patients with or without DM, we investigated the two-year clinical results of patients included in two stent-specific, prospective DES registries.

## Materials and methods

### Study design and population

DM (type 1 or type 2) was defined as either a previous diagnosis of DM treated with pharmacologic or nonpharmacologic measure, or a new DM was defined according to the American Diabetes Association as history of either presence of classic symptoms of DM with unequivocal elevation of plasma glucose (2 h post-prandial or random of ≥200 mg/dL), fasting plasma glucose elevation on ≥126 mg/dl during hospitalization or Hemoglobin A1C ≥6.5% (48 mmol/mol). Patients were considered insulin-treated if they were taking insulin. Patients were considered noninsulin-treated if they were taking only oral hypoglycemic agents or were on a therapeutic lifestyle modification only or both oral agents and therapeutic life-style modification.

The study populations were pooled from two independent, multicenter, all-comer, observational studies of patients undergoing PCI with second-generation DES from of the Korean Registry of Xience V EVERolimus-Eluting coronary STent system (K-EVEREST) and the Clinical Outcomes iN patientS with zoTArolimus-eluting stent implaNTation (CONSTANT) registry. Inclusion criteria, exclusion criteria, and the key features of each registry are summarized in **S1 Table**. Briefly, the K-EVEREST and CONSTANT registries involved prospective, multicenter recruitment of unrestricted patients undergoing PCI with DESs in Korea, and included the use of second-generation DES in contemporary PCI situations. The pooled dataset consisted of individual patient data from two different cohorts of the DES registry.

These registries were supported by the Keimyung University Dongsan Hospital, Deagu, Korea, and there was no industry involvement in the design, conduct, or analysis of the study. The study protocol was approved by the Keimyung University Dongsan Hospital ethics committee/ institutional review boards at each participating center, and all patients provided written informed consent for participation in this prospective registry.

### PCI procedures and clinical follow-up

In the K-EVEREST and CONSTANT registries, PCI procedures were performed according to standard techniques at the discretion of the treating physician. These registries did not specify the stent types based on clinical or anatomic features. Thus, each operator was responsible for the choice of a specific DES. Periprocedural anticoagulants were administered according to standard regimens. Glycoprotein IIb/IIIa inhibitors were administered at the discretion of the operator. All patients undergoing PCI received a loading dose of aspirin and P2Y_12_ receptor inhibitor (clopidogrel, prasugrel, or ticagrelor) before or during PCI. After the procedure, aspirin was continued indefinitely and P2Y_12_ receptor inhibitors were prescribed for at least 12 months, regardless of the DES type. Drugs for secondary prevention were prescribed according to current guidelines.

Clinical follow-up was conducted during hospitalization and at one month, 12 months, and 24 months. At each visit, information pertaining to the patients’ clinical status, all interventions, and outcome events were recorded. Baseline characteristics and outcome data were collected using a dedicated, electronic case report form by specialized personnel at each participating center. The internet-based system provided each center with immediate and continuous feedback on the processes and quality-of-care measures. Monitoring and verification of registry data were periodically performed at the participating hospitals by members of the academic coordinating center (Clinical Research Center, Keimyung University Dongsan Hospital, Deagu, Korea).

### Study outcomes and definitions

The primary clinical outcomes were major cardiac adverse events (MACE), composite endpoints of death from any cause, myocardial infarction (MI), any repeat revascularization. Secondary clinical outcomes included death (cardiac or non-cardiac), MI (Q-wave or non-Q-wave), repeat revascularization, and stent thrombosis.

Death was considered to have a cardiac cause unless an unequivocal non-cardiac cause could be established. The protocol definition of MI was pre-specified and was based on the universal definition of MI [8]. MI was defined as any increase in cardiac enzymes above the upper range limit with or without the development of Q waves on the electrocardiogram and peri-procedural MI was excluded from the analysis. Repeat revascularization included any type of percutaneous or surgical revascularization procedures and was categorized as revascularization of any lesion, target lesion, or target vessel. Definite stent thrombosis was assessed according to the Academic Research Consortium definition [9]. All outcomes of interest were confirmed by source documentation collected at each hospital and were carefully verified and adjudicated by independent clinicians at each hospital.

### Statistical analysis

Based on a history of DM, the data were reported as frequencies and percentages for dichotomous and categorical variables, and as the mean ± standard deviation for continuous variables. Dichotomous and categorical variables were assessed using Chi-square tests and Fisher’s exact tests, and continuous variables were assessed using Student’s t tests or the Wilcoxon rank-sum tests, as appropriate. Observed event rates at 2-year and survival curves were generated using the Kaplan-Meier method and compared with the log-rank test. For clinical outcomes, we prespecified examining the event rates 2-year of follow-up, and separately from 0 to 1 years and from 1 to 2 years (as the landmark analyses). We further classified the 0–1 year period as the early period and the 1–2 year period as the late period. Univariate and multivariate analyses of hazard ratios, including the baseline covariates in Tables 1 and 2, were calculated using the Cox proportional hazard method. Factors with p values <0.1 in the univariate analysis were entered into the multivariate model. In addition, unadjusted and adjusted Cox proportional hazard models were used to compare clinical events according to a history of DM. To compensate for the nonrandomized design of the observational studies and to reduce the effect of potential confounding factors on the outcomes, we used a propensity score method. We fitted weighted Cox proportional hazards models using the inverse probability of treatment weighting (IPTW) [10].

**Table 1:** Baseline demographic and clinical characteristics of patients.

Characteristics	Non-DM (n = 1308)	DM (n = 605)	P Value	SMD
Age (years)	63.7 ± 12.4	64.8 ± 10.1	0.037	0.119
Men	948 (72.5%)	382 (63.1%)	<0.001	0.201
Body-mass index (kg/m^2^)	24.3 ± 3.1	24.6 ± 3.4	0.12	0.077
Hypertension	638 (48.8%)	401 (66.3%)	<0.001	0.360
Hyperlipidemia	232 (17.7%)	149 (24.6%)	0.001	0.169
Current smoker	490 (37.5%)	190 (31.4%)	0.012	0.128
Atrial fibrillation	29 (2.2%)	14 (2.3%)	0.99	0.007
Previous MI	24 (1.8%)	12 (2.0%)	0.97	0.011
Previous PCI	105 (8.0%)	69 (11.4%)	0.021	0.114
Previous CABG	18 (1.4%)	8 (1.3%)	0.99	0.005
Renal failure	76 (5.8%)	77 (12.7%)	<0.001	0.270
Cerebrovascular disease	19 (1.5%)	41 (6.8%)	<0.001	0.240
Ejection fraction (%)	57.4 ± 10.9	55.5 ± 11.8	0.001	0.161
Clinical presentation			0.007	0.155
Stable angina	390 (29.8%)	201 (33.2%)		
Unstable angina	363 (27.8%)	190 (31.4%)		
NSTEMI	259 (19.8%)	117 (19.3%)		
STEMI	296 (22.6%)	97 (16.0%)		
Discharge medications				
Aspirin	1294 (98.9%)	593 (98.0%)	0.16	0.074
ADP receptor antagonist	1219 (93.2%)	554 (91.6%)	0.24	0.061
Cilostazol	211 (16.1%)	142 (23.5%)	<0.001	0.185
β-blocker	989 (75.6%)	434 (71.7%)	0.08	0.088
Calcium channel blocker	226 (17.3%)	125 (20.7%)	0.087	0.086
ACE inhibitor or ARB	859 (65.7%)	395 (65.3%)	0.91	0.008
Statin	1063 (81.3%)	491 (81.2%)	0.99	0.003

Data are shown as mean (SD) for continuous variables and absolute numbers (percentage) for dichotomous variables.

Abbreviations: CABG, coronary artery bypass grafting; CAD, coronary artery disease; CHF, congestive heart failure; NSTEMI, non-ST-elevation myocardial infarction; MI, myocardial infarction; PCI, percutaneous coronary intervention; STEMI, ST-elevation MI; SMD, standardization mean differences

**Table 2:** Baseline lesion and procedural characteristics.

Characteristics	Non-DM (n = 1794)	DM (n = 820)	P Value
Treated lesion			0.048
LM	76 (4.2%)	37 (4.5%)	
LAD	811 (45.2%)	386 (47.1%)	
LCX	345 (19.2%)	182 (22.2%)	
RCA	562 (31.3%)	215 (26.2%)	
ACC–AHA lesion type			0.98
A	40 (2.2%)	18 (2.2%)	
B1	388 (21.6%)	183 (22.3%)	
B2	363 (20.2%)	162 (19.8%)	
C	1003 (55.9%)	457 (55.7%)	
Restenotic lesions	39 (2.2%)	21 (2.6%)	0.64
Moderate to severe CAC	176 (9.8%)	85 (10.4%)	0.71
Bifurcation lesions	330 (18.4%)	167 (20.4%)	0.26
Ostial lesion	126 (7.0%)	67 (8.2%)	0.34
Thrombus present	215 (12.0%)	86 (10.5%)	0.29
Chronic total occlusion	98 (5.5%)	44 (5.4%)	0.99
Lesion length (mm)	23.6 ± 12.6	23.7 ± 12.2	0.92
Proximal RVD (mm)	3.1 ± 0.5	3.1 ± 0.5	0.69
Distal RVD (mm)	2.8 ± 0.5	2.8 ± 0.5	0.59
Diameter stenosis (%)	83.7 ± 10.4	83.4 ± 10.5	0.63
Pre-balloon dilatation	1606 (89.5%)	748 (91.2%)	0.20
Post-high pressure NC balloon	534 (29.8%)	271 (33.0%)	0.10
No. of treated lesion per patients	1.4 ± 0.6	1.4 ± 0.6	0.57
No. of stents per patient	1.6 ± 0.9	1.7 ± 0.9	0.001
No. of stents per lesion	1.2 ± 0.4	1.2 ± 0.4	0.30
Stent diameter (mm) per patient	3.1 ± 0.4	3.1 ± 0.4	0.99
Stent diameter (mm) per lesion	3.1 ± 0.4	3.1 ± 0.4	0.99
Stent length (mm) per patient	36.7 ± 23.2	40.8 ± 24.7	<0.001
Stent length (mm) per lesion	28.1 ± 13.9	27.7 ± 13.2	0.52
Type of DES			0.53
Everolimus-Eluting	922 (51.4%)	433 (52.8%)	
Zotarolimus-Eluting	872 (48.6%)	387 (47.2%)	
Peri-procedure related MI*	4 (0.3%)	2 (0.3%)	0.99

Data are shown as mean (SD) for continuous variables and absolute numbers (percentage) for dichotomous variables.

* Peri-procedure related MIs were calculated per patient.

Abbreviations: LAD, left anterior descending artery; LCX, left circumflex artery; LM, left main; RCA, and right coronary artery; CAC, coronary artery calcification; RVD, reference vessel diameter; NC, non-compliant. Other abbreviations are as in Table 1.

All reported P values were 2-sided and have not been adjusted for multiple testing. All analyses were performed with SPSS software version 22.0 (SPSS Inc., Chicago, Illinois) and the R programming language.

## Results

### Characteristics of the study patients

During February 2009 and December 2013, a total of 1,913 patients from two stent-specific, prospective K-EVERST and CONSTANT registries were included in the current study (**S1 Fig**). The baseline demographics and clinical characteristics of the study population according to the patients’ DM status are shown in **Table 1**. The mean age of the patients was 63.9 years and approximately 70% of patients were men. Patients with DM were older, included more men, and had higher rates of hypertension, hyperlipidemia, histories of previous PCI, renal failure, and cerebrovascular disease than patients without DM. There was no difference in discharge medication between the two groups, except for cilostazol. **Table 2** shows the lesion and procedural characteristics of the study population according to patients’ DM status. The number of stents was higher and the stent length was longer in patients with DM. **Table 3** shows the univariate and multivariate analyses of MACE and TVR. DM and renal failure had the highest correlation with 2-year clinical outcomes in multivariate analyses. However, the type of DES was not a significant predictor of 2-year clinical outcomes.

**Table 3:** Univariate and multivariate Cox proportional hazard analyses for MACE and TVR in enrolled patients.

MACE†					Target-Vessel Revascularization				
	Univariate		Multivariate			Univariate		Multivariate	
Variables	HR (95% CI)	P	HR (95% CI)	P	Variables	HR (95% CI)	P	HR (95% CI)	P
Overall					Overall				
Renal failure	4.89 (2.99–8.00)	<0.001	3.61 (2.18–6.00)	<0.001	In-stent restenosis	3.59 (1.54–8.37)	0.003	3.97 (1.69–9.33)	0.002
Previous CABG	2.83 (1.16–6.91)	0.022	2.94 (1.20–7.17)	0.018	Renal failure	3.37 (1.35–8.43)	0.009	2.84 (1.11–7.28)	0.030
Diabetes mellitus	2.24 (1.63–3.08)	<0.001	1.97 (1.42–2.74)	<0.001	Ostial lesion	2.03 (1.02–4.00)	0.042	2.30 (1.16–4.56)	0.017
Left main	1.69 (0.98–2.93)	0.061	1.63 (0.94–2.84)	0.081	Left main	2.64 (1.25–5.58)	0.011	2.25 (1.02–4.96)	0.045
Previous CVA	1.53 (0.93–2.54)	0.097	*		Diabetes mellitus	1.98 (1.18–3.31)	0.010	1.87 (1.10–3.17)	0.021
ACC-AHA B2/C lesion	1.27 (1.03–1.57)	0.028	*		Bifurcation	0.84 (0.44–1.63)	0.615	*	
Stent length (per 1-mm)	1.01 (1.00–1.01)	0.066	*		Previous PCI	1.83 (0.90–3.72)	0.097	*	
Bifurcation	0.89 (0.60–1.33)	0.586	*		Cilostazol	1.61 (0.89–2.90)	0.095	*	
Statin	0.71 (0.49–1.03)	0.069	*		†			*	
**0–1 year**					**0–1 year**				
Renal failure	6.00 (3.33–10.82)	<0.001	3.95 (2.15–7.26)	<0.001	Renal failure	4.44 (1.57–12.57)	0.005	2.99 (1.03–8.65)	0.043
Previous CABG	4.74 (1.92–11.68)	0.001	4.19 (1.69–10.39)	0.002	Diabetes mellitus	3.37 (1.72–6.63)	<0.001	3.08 (1.55–6.15)	0.001
Diabetes mellitus	3.34 (2.17–5.12)	<0.001	2.77 (1.78–4.33)	<0.001	†				
Previous CVA	2.12 (1.17–3.82)	0.013	*		†				
ACC-AHA B2/C lesion	1.28 (0.96–1.70)	0.088	*		†				
**1–2 year**					**1–2 year**				
Renal failure	3.32 (1.33–8.26)	0.010	3.58 (1.43–8.95)	0.006	In-stent restenosis	11.3 (4.46–28.7)	<0.001	10.6 (4.19–27.1)	<0.001
In-stent restenosis	3.33 (1.44–7.72)	0.005	3.55 (1.53–8.26)	0.003	Ostial lesion	3.42 (1.35–8.68)	0.010	3.21 (1.21–8.55)	0.019
Left main	2.38 (1.13–4.98)	0.022	2.24 (1.06–4.70)	0.033	Cilostazol	2.49 (1.05–5.87)	0.037	*	
†					Left main	3.46 (1.18–10.17)	0.024	*	
†					Clopidogrel	0.38 (0.13–1.12)	0.080	0.31 (0.10–0.94)	0.039

*Not retained as independent predictor in multivariate analysis.

†Not significant in univariate analysis.

Abbreviations: CHF, congestive heart failure; CI, confidence interval; HR, hazard ratio; MACE, major adverse cardiac event; TVR, target-vessel revascularization. Other abbreviations are as in Tables 1 and 2.

### Clinical impact of diabetes mellitus

**Fig 1:**
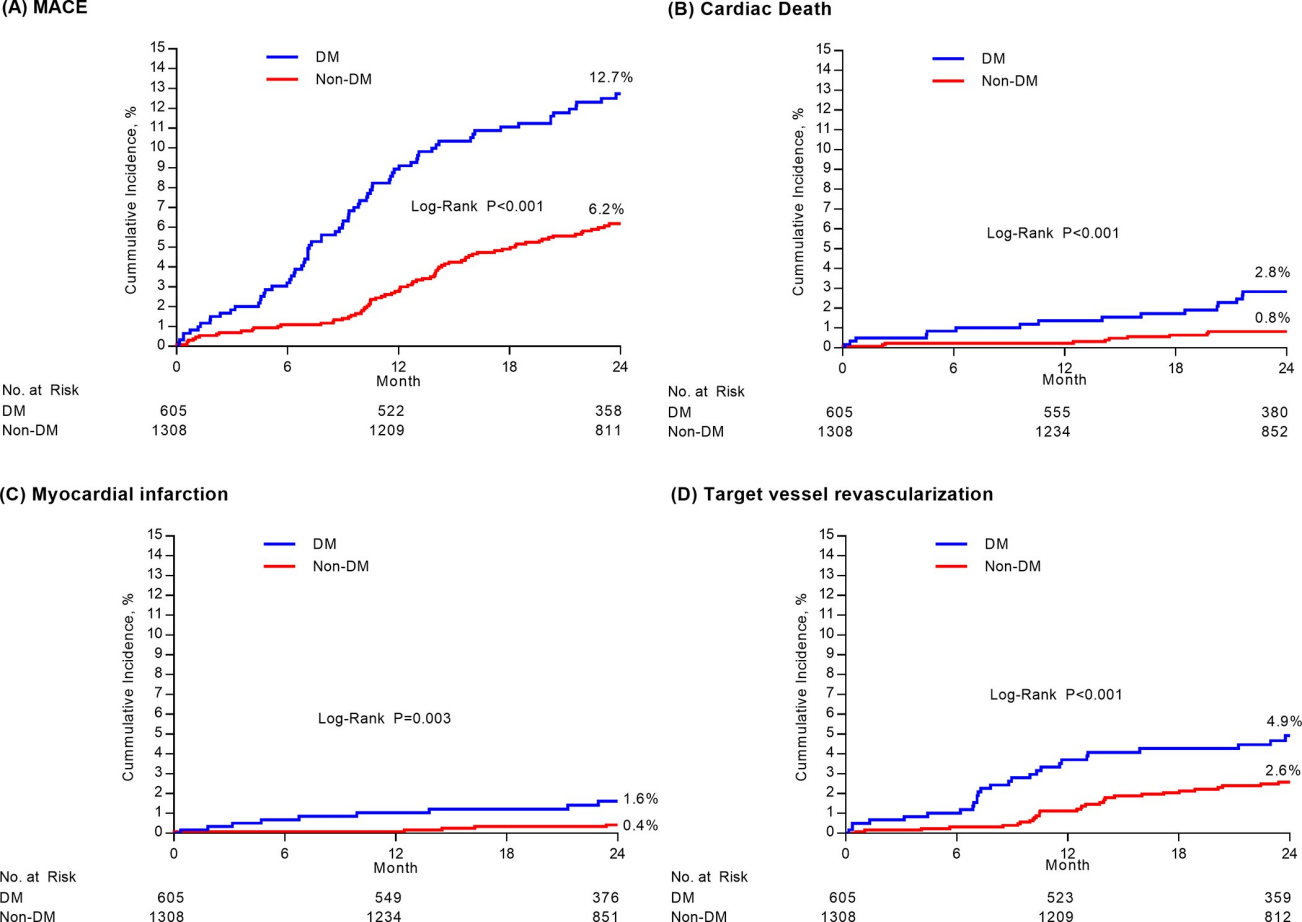
Kaplan-Meier curve for 2 years clinical outcomes according to diabetes mellitus. In each figure, cumulative-incidence curves are shown for major adverse cardiac events (MACE) stratified by diabetes mellitus (Panel A), cardiac death (Panel B), myocardial infarction (Panel C), and target vessel revascularization (Panel D). MACE was defined as a composite of death from any causes, myocardial infraction, or repeat revascularization.

The median duration of clinical follow-up in the overall population was 2.0 years (interquartile range 1.9–2.1). The baseline characteristics after IPTW adjustment are shown in the **S2 Table**. The Kaplan–Meier estimates of primary and secondary outcomes at 2-years according to DM status are shown in **Table 4 and Fig 1**. The incidence of 2-year MACE was significantly higher in patients with DM than in those without DM (12.7% vs. 6.2%, P<0.001). Death, MI, and repeat revascularization showed significantly higher rates in patients with DM than in those without DM. After IPTW adjustment, patients with DM also showed higher event rates of primary and secondary endpoints, including cardiac death, MI, and repeat revascularization than patients without DM. In addition, the impact of diabetes mellitus according to clinical and procedural subgroups is shown in **S2 Fig**.

**Table 4:** Hazard ratios of 2-year event rates of clinical outcomes according to diabetes mellitus.

				Unadjusted		IPTW adjusted	
Characteristics	Non-DM (n = 1308)	DM (n = 605)	P Value	Hazard Ratio (95% CI)	P Value	Hazard Ratio (95% CI)	P Value
MACE	76 (6.2)	75 (12.7)	<0.001	2.24 (1.63–3.08)	<0.001	2.07 (1.50–2.86)	<0.001
Death from any cause	23 (1.9)	31 (5.2)	<0.001	2.97 (1.73–5.09)	<0.001	2.21 (1.27–3.83)	0.005
Cardiac death	10 (0.8)	17 (2.8)	<0.001	3.74 (1.71–8.17)	0.001	2.42 (1.11–5.27)	0.026
Non-cardiac death	13 (1.1)	14 (2.5)	0.021	2.37 (1.11–5.05)	0.025	2.02 (0.92–4.40)	0.078
Myocardial infarction	5 (0.4)	10 (1.6)	0.003	4.43 (1.51–12.95)	0.007	4.71 (1.63–13.63)	0.004
Q wave MI	2 (0.2)	5 (0.7)	0.022	5.51 (1.07–28.42)	0.041	5.74 (1.15–28.57)	0.033
Non-Q wave MI	3 (0.3)	5 (0.9)	0.055	3.70 (0.88–15.49)	0.073	3.98 (0.96–16.61)	0.058
Repeat revascularization	56 (4.6)	47 (8.2)	<0.001	1.91 (1.30–2.81)	0.001	2.07 (1.41–3.03)	<0.001
Target vessel	31 (2.6)	27 (4.9)	<0.001	1.98 (1.18–3.31)	0.010	1.94 (1.16–3.26)	0.012
Target lesion	22 (3.9)	19 (5.2)	0.029	1.96 (1.06–3.62)	0.032	1.81 (0.95–3.42)	0.071
Non-target vessel	25 (2.1)	20 (3.5)	0.042	1.83 (1.01–3.29)	0.045	2.22 (1.26–3.91)	0.006
Definite or probable stent thrombosis	3 (0.2)	3 (0.5)	0.32	2.2 (0.44–10.91)	0.33	2.52 (0.53–12.03)	0.25

*Cumulative rates of events based on Kaplan-Meier estimates.

Abbreviations: DM, diabetes mellitus; CI, confidence interval; IPTW, inverse probability of treatment weighting; MACE, major adverse cardiac event; MI, myocardial infarction.

### Landmark analyses of clinical outcomes

**Fig 2:**
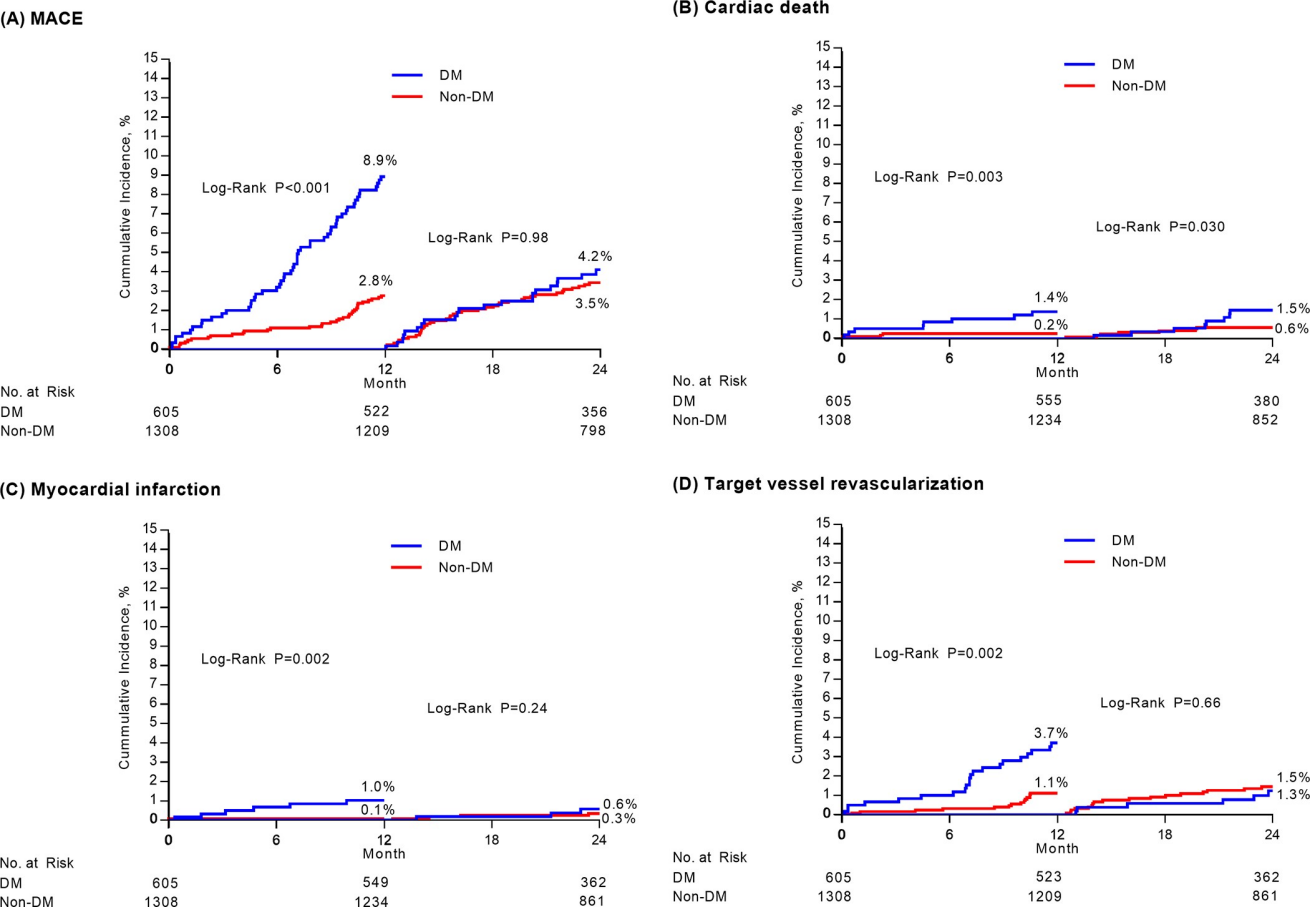
Kaplan-Meier landmark curves between 0 and 1 years and 1 and 2 years according to diabetes mellitus. In each figure, cumulative-incidence curves are shown for major adverse cardiac events (MACE) stratified by diabetes mellitus (Panel A), cardiac death (Panel B), myocardial infarction (Panel C), and target vessel revascularization (Panel D). MACE was defined as a composite of death from any causes, myocardial infraction, or repeat revascularization.

A landmark analysis (**Table 5 and Fig 2**) after IPTW adjustment revealed that, the incidences of MACE (2.8% vs. 8.9%, HR 3.04, 95% CI; 1.97–4.68; P <0.001), cardiac death, MI, and repeat revascularization in the early (0–1 year) period were significantly higher in patients with DM than those without DM. However, these findings were not observed in the late period (1–2 year). The incidence of MACE (3.5% vs. 4.2%, HR 1.24, 95% CI; 0.74–2.07; P = 0.41), cardiac death, MI, and repeat revascularization in the late period were not statistically different between patients with and without DM.

**Table 5:** Landmark analysis of 1-year and 1- to 2 year event rates of clinical outcomes according to diabetes mellitus.

	MACE During 1 year Follow-up				MACE During 1- to 2 year Follow-up			
Characteristics	Crude Event rate (%)		IPTW adjusted Hazard Ratio (95% CI)	P Value	Crude Event rate (%)		IPTW adjusted Hazard Ratio (95% CI)	P Value
	Non-DM	DM			Non-DM	DM		
MACE	35 (2.8)	52 (8.9)	3.04 (1.97–4.68)	<0.001	41 (3.5)	23 (4.2)	1.24 (0.74–2.07)	0.41
Death from any cause	9 (0.7)	18 (3.1)	3.04 (1.34–6.90)	0.008	14 (1.2)	13 (2.2)	1.67 (0.78–3.57)	0.19
Cardiac death	3 (0.2)	8 (1.4)	4.03 (1.11–14.57)	0.034	7 (0.6)	9 (1.5)	1.74 (0.63–4.78)	0.29
Non-cardiac death	6 (0.5)	10 (1.7)	2.48 (0.85–7.26)	0.097	7 (0.6)	4 (0.7)	1.59 (0.50–5.03)	0.43
Myocardial infarction	1 (0.1)	6 (1.0)	15.73 (1.82–135.7)	0.012	4 (0.3)	4 (0.6)	2.13 (0.53–8.58)	0.29
Q wave MI	1 (0.1)	1 (0.2)	3.40 (0.27–45.04)	0.35	1 (0.1)	4 (0.6)	7.72 (0.94–63.10)	0.057
Non-Q wave MI	0 (0.0)	5 (0.9)	-	-	3 (0.3)	0 (0.0)	-	-
Repeat revascularization	26 (2.1)	34 (6.0)	3.02 (1.82–5.02)	<0.001	30 (2.6)	13 (2.4)	1.21 (0.65–2.24)	0.54
Target vessel	14 (1.1)	21 (3.7)	3.16 (1.61–6.20)	<0.001	17 (1.5)	6 (1.3)	0.86 (0.34–2.17)	0.75
Target lesion	8 (0.6)	15 (2.7)	3.70 (1.50–9.10)	0.005	14 (1.2)	4 (0.8)	0.73 (0.25–2.16)	0.57
Non-target vessel	12 (1.0)	13 (2.3)	2.85 (1.32–6.18)	0.008	13 (1.1)	7 (1.2)	1.66 (0.71–3.86)	0.24
Definite or probable Stent thrombosis	1 (0.1)	2 (0.3)	5.26 (0.48–58.06)	0.18	2 (0.2)	1 (0.2)	1.23 (0.12–12.47)	0.86

*Cumulative rates of events based on Kaplan-Meier estimates.

Abbreviations: DM, diabetes mellitus; CI, confidence interval; IPTW, inverse probability of treatment weighting; MACE, major adverse cardiac event; MI, myocardial infarction.

## Discussion

The major findings from the analyses of two well-managed registries for the impact of DM on clinical outcomes of patients treated with second-generation DES are (1) that, despite the second-generation DES era, DM was still an independent factor for increased adverse cardiac events; (2) that most MACE occurred in the early (0–1 year) period, and DM did not affect the events in the late (1–2 year) period; and (3) that, in multivariate analyses, DM was an independent predictor of MACE and TVR in the early period, but not in the late period.

### Previous studies of DES in diabetes mellitus

The clinical outcomes of the second-generation DES in patients with DM have been evaluated and early findings showed that the second-generation DES in DM patients had better clinical outcomes than the first-generation DES. The use of EES, second-generation DES, reduced the 8-month angiographic restenosis rates of MI, death, or stent thrombosis compared to implantation with sirolimus-eluting stent (SES) in patients with DM and coronary artery disease in the ESSENCE-DIABETES (Randomized Comparison of EES Versus SES Implantation for De Novo Coronary Artery Disease in Patients With Diabetes Mellitus) trial [11].

In contrast, recent findings that second-generation DES had no difference in clinical outcomes compared to first-generation DES have also been reported in diabetes patients. In the SPIRIT (Clinical Evaluation of the XIENCE V Everolimus Eluting Coronary Stent System) IV randomized trial, comparison of EESs with PES showed no difference in 1-year target-lesion failure in diabetic patients (6.4% vs. 6.9%, p = 0.80) [12]. And another recent patient-level pooled analysis from two large-scale prospective multicenter randomized trials according to the presence of DM found no significant differences in the rates of 3-year all-cause death, MI, any TLR (DM stratum, 10.1% vs. 8.7%, P = 0.23; and non-DM stratum, 6.2% vs. 5.7%, P = 0.62), or stent thrombosis [13]. Our study using stent-specific, clinical registries of diverse types of second-generation DES in routine clinical practice also found similar clinical outcomes to recent studies and may provide important information that DM is a still strong clinical risk factor in the second-generation DES era.

### Differences in clinical outcomes of diabetic versus non-diabetic patients between early and late periods

In the current study, there was a difference in MACE between DM patients and non-DM patients in the early period, and it was driven mainly by target-vessel repeat revascularization. And TVR events in our study most commonly occurred within 6–9 months after DES implantation. This phenomenon might be due to neointimal growth in the several months following DES implantation, and follow-up angiography at around 9 months. In another large cohort study, similar to our data, clinical events occurred mainly in DM patients at 6–9 months, and the TVR rate was 3.5%, which are in line with our results [14]. A previous animal study supported the hypothesis that delayed neointimal hyperplasia and inflammation may cause early aggravation of neo-atherosclerosis in patients with DM [15]. Therefore, the difference in clinical outcomes between the DM and non-DM patients in the early period may have been caused by the time required for healthy neointima to completely cover the stent strut.

In the late period, interestingly, most clinical outcomes did not show any difference between patients with or without DM. In our data, the occurrence of MACE was 4.2% in the DM group and 3.5% in the non-DM group in the late period, and there was no significant difference between the groups. After IPTW adjustment, the TVR rate was slightly lower in DM patients compared to the non-DM group (1.3% in the DM group and 1.5% in the non-DM group, HR 0.86, 95% CI; 0.34–2.17; P = 0.75) in the late period. This trend is plausible because the neoinimal growth or thrombogenic material is more active near the stent strut in the early period after implantation and thus, it could be affected by the type of immunosuppressive drug, platform design or polymer in a DES. In contrast, beyond 1-year, the rapid reaction of neointimal growth or thrombogenic material near the stent would have subsided and be less affected by the differences in the DES components. Thus, even between DES and bare metal stent, the long-term clinical outcome after 1-year would not be different in previous several studies [4, 16]. The rapid decrease in repeat revascularizations in the late period has also been observed in several previous studies and trials related to DM [17, 18]. And, our current study showed a difference in the clinical outcomes in the early and late periods in DM patients through landmark analysis, similar to the previous study.

### Predictor of the clinical outcome according to period after DES implantation

In multivariate analyses of the current cohort registry, patient factors, such as DM and chronic renal failure, were found to be independent predictors of MACE and TVR in the early period. On the other hand, anatomical and procedural factors, such as in-stent restenosis and ostial lesions, were found to independent predictors of MACE and TVR in the late period, and this finding was similar to that reported by another study [18]. As mentioned previously, patient factors were still important risk factors in the second generation DES era in the early period. Our results provide useful information to physicians and suggest that more intensive cardiac evaluation and management of risk factors in the early period following DES implantation may be helpful for DM patients. However, follow-up strategies of 1-year after DES implantation in DM patients may be acceptable without any difference from non-DM patients. In the late period, intensive cardiac evaluation may be helpful in patients with complex procedural factors. In addition, the listed DM, chronic renal failure, in-stent restenosis, and ostial lesions were associated with worse outcomes following second generation DES implantation, so coronary artery bypass grafting (CABG) options should always be considered in treatment planning.

### Study limitations

Several limitations of our study should be considered. First, as this study was observational in nature, the overall findings should be considered hypothetical and hypotheses-generating only. Second, analysis of clinical outcomes was limited to the 2-year period after the index PCI because of the study protocol. Our study was not able to draw any conclusions regarding very long-term prognoses beyond 2-years in diabetic patients. Third, because the data were from observational registries, the clinical events may not have been meticulously captured, and patient follow-up may not have been as strict as would have been in a randomized trial. This may have been the reason for the low event rates seen in this study, especially the rate of target MI, which was much lower in our study than in the previous randomized controlled trial. We cannot completely exclude the possibility of under-investigating of clinical outcomes, such as MI, TVR, or ST, in the patients lost to follow-up. However, our data showed a relatively high follow-up rate compared to other registries, which is a meaningful value.

## Conclusions

In the real-world practice using second-generation DES, DM significantly increased the 2-year event rate of MACE, mainly due to clinical events in the early period (0–1 year). Therefore, in DM patients, we advise intentional cardiac evaluation and management of risk factors in the early period after PCI with second-generation DES.

## Supporting information

S1 Fig Key features of each stent-specific registry. Abbreviations: DES, drug-eluting stents; PCI, percutaneous coronary intervention.
(TIF)

S2 Fig Impact of diabetes mellitus across clinical and procedural subgroups. Abbreviations: MACE, major adverse cardiac event; TVR, target vessel revascularization.
(TIF)

S1 Table Key features of each stent-specific registry. Abbreviations: DES, drug-eluting stents; DS, diameter stenosis; CoCr-EES, cobalt-chromium everolimus-eluting stent(s); IQR, interquartile range; Re-ZES, Resolute zotarolimus-eluting stent(s).
(PDF)

S2 Table Adjusted baseline characteristics of patients using inverse probability weighting. Data are shown as mean (SD) for continuous variables and absolute numbers (percentage) for dichotomous variables. Abbreviations: CABG, coronary artery bypass grafting; CAD, coronary artery disease; CHF, congestive heart failure; NSTEMI, non-ST-elevation myocardial infarction; MI, myocardial infarction; PCI, percutaneous coronary intervention; STEMI, ST-elevation MI; SMD, standardization mean differences.
(PDF)

S1 Data (CSV)

## Abbreviations

DES: drug-eluting stent
DM: diabetes mellitus
EES: everolimus-eluting stent
IPTW: inverse probability of treatment weighting
MACE: major cardiac adverse events
MI: myocardial infarction
PES: paclitaxel-eluting stent
PCI: percutaneous coronary interventions
SES: sirolimus-eluting stent
ST: stent thrombosis
TLR: target lesion revascularization
TVR: target vessel revascularization
ZES: zotalolimus-eluting stent
