# Supplementary figures and images for: Clinical impact of diabetes mellitus on 2-year clinical outcomes following PCI with second-generation drug-eluting stents; Landmark analysis findings from patient registry: Pooled analysis of the Korean multicenter drug-eluting stent registry
Source: PLoS One. 2020 Jun 10;15(6):e0234362. doi: 10.1371/journal.pone.0234362 (PMC7286514; doi:10.1371/journal.pone.0234362)

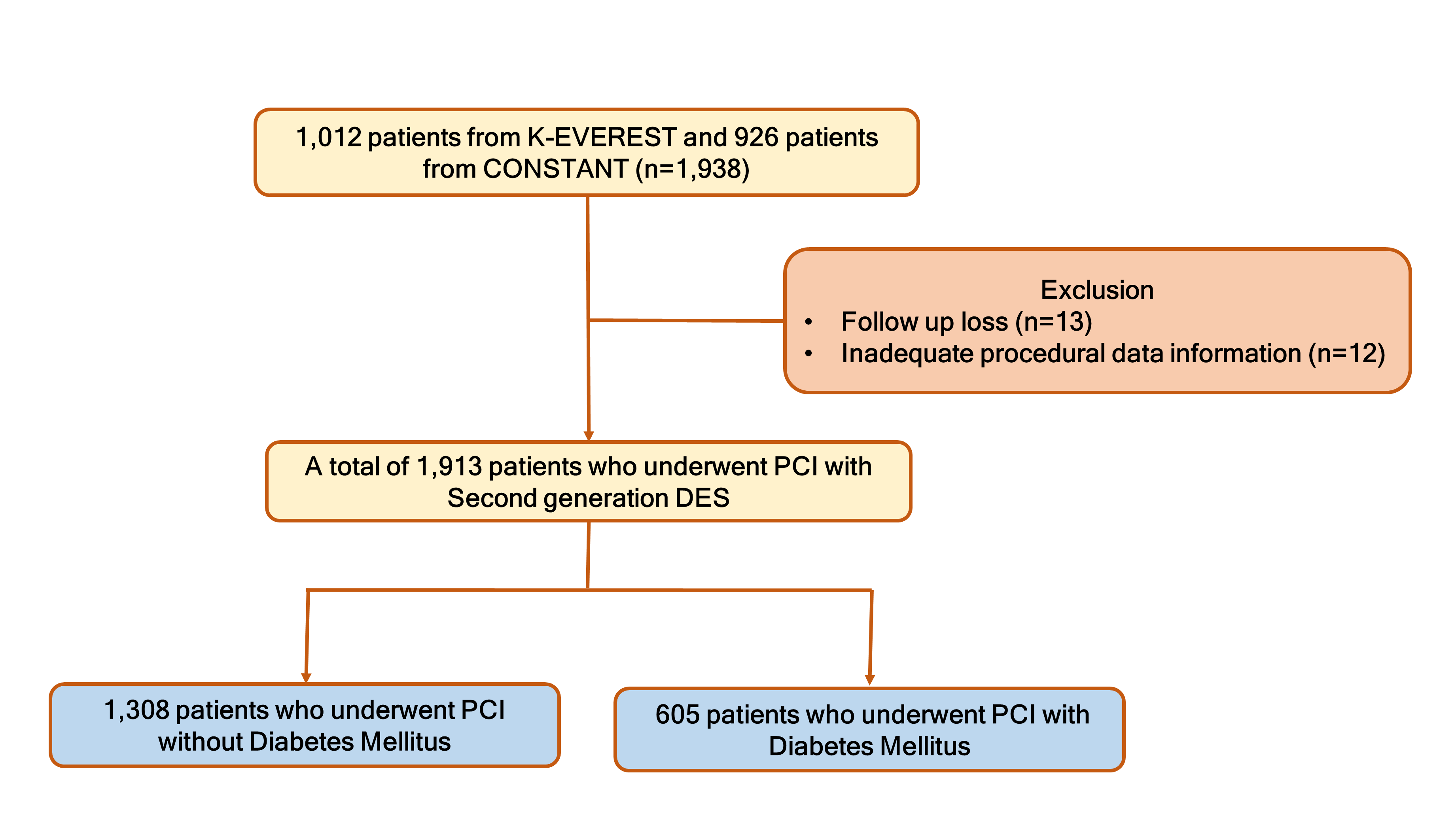

Supplement: S1 Fig — Abbreviations: DES, drug-eluting stents; PCI, percutaneous coronary intervention. (TIF) [file pone.0234362.s001.tif]

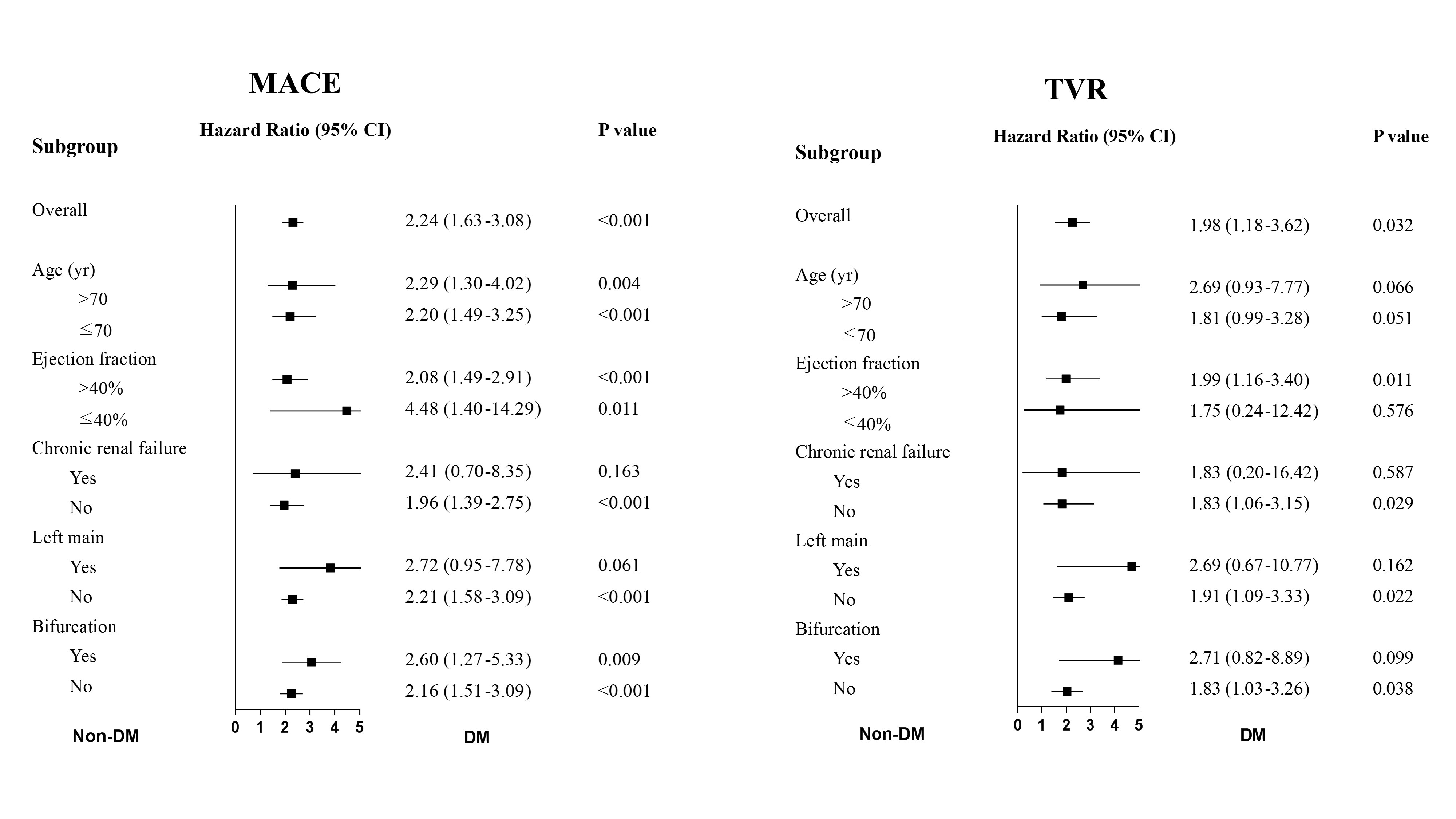

Supplement: S2 Fig — Abbreviations: MACE, major adverse cardiac event; TVR, target vessel revascularization. (TIF) [file pone.0234362.s002.tif]
