# Supplementary material for: Clinical impact of diabetes mellitus on 2-year clinical outcomes following PCI with second-generation drug-eluting stents; Landmark analysis findings from patient registry: Pooled analysis of the Korean multicenter drug-eluting stent registry
Source: PLoS One. 2020 Jun 10;15(6):e0234362. doi: 10.1371/journal.pone.0234362 (PMC7286514; doi:10.1371/journal.pone.0234362)
Supplement: S1 Table — Abbreviations: DES, drug-eluting stents; DS, diameter stenosis; CoCr-EES, cobalt-chromium everolimus-eluting stent(s); IQR, interquartile range; Re-ZES, Resolute zotarolimus-eluting stent(s). (PDF) [file pone.0234362.s003.pdf]

Design	K-EVEREST	CONSTANT
Study type	21 Centers, prospective, nonrandomized observational registry including PCI with CoCr-EES	7 Centers, prospective, nonrandomized observational registry including PCI with Re-ZES
Stent name	Xience V™	Resolute-Integrity™
Stent type used for PCI	Cobalt-chromium everolimus-eluting stents	Zotarolimus-eluting stents
Main inclusion criteria	DS for epicardial coronary artery ≥70%, or 50%–70% with objective evidence of myocardial ischemia; diameter stenosis for left main coronary artery ≥50%, and amenable to PCI.	
Exclusion criteria	The exclusion criteria were minimal. Patients with cardiogenic shock, malignant disease, or other comorbid conditions with a life expectancy of <12 months; those treated with a mixture of different types of DES; and those with planned surgery necessitating interruption of antiplatelet drugs within 6 months after the procedure were excluded.	
Recruitment period	Feb 2009–Apr 2011	Jul 2011–Dec 2013
Follow-up period (median and IQR), y	2.03 (1.96–2.08)	2.02 (1.97–2.07)

Abbreviations: DES, drug-eluting stents; DS, diameter stenosis; CoCr-EES, cobalt-chromium everolimus-eluting stent(s); IQR, interquartile range; Re-ZES, Resolute zotarolimus-eluting stent(s).
