# Supplementary material for: Clinical impact of diabetes mellitus on 2-year clinical outcomes following PCI with second-generation drug-eluting stents; Landmark analysis findings from patient registry: Pooled analysis of the Korean multicenter drug-eluting stent registry
Source: PLoS One. 2020 Jun 10;15(6):e0234362. doi: 10.1371/journal.pone.0234362 (PMC7286514; doi:10.1371/journal.pone.0234362)
Supplement: S2 Table — Data are shown as mean (SD) for continuous variables and absolute numbers (percentage) for dichotomous variables. Abbreviations: CABG, coronary artery bypass grafting; CAD, coronary artery disease; CHF, congestive heart failure; NSTEMI, non-ST-elevation myocardial infarction; MI, myocardial infarction; PCI, percutaneous coronary intervention; STEMI, ST-elevation MI; SMD, standardization mean differences. (PDF) [file pone.0234362.s004.pdf]

Characteristics	Non-DM (n=1309.1)	DM (n=602.5)	SMD
Age (years)	64.02 (11.14)	64.16 (10.08)	0.013
Men	0.70 (0.46)	0.70 (0.46)	0.002
Body-mass index (kg/m^2)	24.41 (3.15)	24.41 (3.23)	0.001
Hypertension	0.55 (0.50)	0.55 (0.50)	0.01
Hyperlipidemia	0.20 (0.40)	0.20 (0.40)	0.012
Current smoker	0.35 (0.48)	0.35 (0.48)	0.017
Atrial fibrillation	0.02 (0.15)	0.02 (0.14)	0.014
Previous MI	0.02 (0.14)	0.02 (0.15)	0.022
Previous PCI	0.09 (0.28)	0.09 (0.29)	0.004
Previous CABG	0.01 (0.11)	0.01 (0.11)	0.009
Renal failure	0.03 (0.18)	0.03 (0.18)	0.002
Cerebrovascular disease	0.08 (0.27)	0.08 (0.27)	0.001
Ejection fraction (%)	56.8 (10.7)	56.94 (10.5)	0.011
Clinical presentation			0.001
Stable angina	0.31 (0.46)	0.31 (0.46)	
Unstable angina	0.29 (0.45)	0.29 (0.46)	
NSTEMI	0.20 (0.40)	0.20 (0.40)	
STEMI	0.20 (0.40)	0.20 (0.40)	
Discharge medications			
Aspirin	0.99 (0.11)	0.99 (0.12)	0.007
ADP receptor antagonist	0.93 (0.26)	0.93 (0.25)	0.018

Cilostazol	0.19 (0.39)	0.19 (0.39)	0.002
β-blocker	0.75 (0.43)	0.74 (0.44)	0.01
Calcium channel blocker	0.18 (0.39)	0.19 (0.39)	0.008
ACE inhibitor or ARB	0.66 (0.47)	0.65 (0.48)	0.01
Statin	0.81 (0.39)	0.81 (0.40)	0.009

Data are shown as mean (SD) for continuous variables and absolute numbers (percentage) for dichotomous variables. Abbreviations: CABG, coronary artery bypass grafting; CAD, coronary artery disease; CHF, congestive heart failure; NSTEMI, non-ST-elevation myocardial infarction; MI, myocardial infarction; PCI, percutaneous coronary intervention; STEMI, ST-elevation MI; SMD, standardization mean differences
